# Synchronous metastatic cutaneous squamous cell carcinoma and chronic lymphocytic leukaemia/small lymphocytic lymphoma in a cervical lymph node: Case report of an unusual event

**DOI:** 10.4317/jced.52643

**Published:** 2015-12-01

**Authors:** Harim-Tavares dos Santos, Bruno-Augusto Benevenuto, Edson-Robles-Castilla Filho, Albina Altemani

**Affiliations:** 1 DDS, MSc. Oral Pathology Section, Department of Oral Diagnosis, Piracicaba Dental School, University of Campinas (UNICAMP), Av. Limeira, Piracicaba, Brazil; 2 DDS, PhD. Oral Pathology, School of Dentistry, Rio de Janeiro Federal University, Rio de Janeiro, Brazil; 3 MD, Student. Department of Pathology, Medical Sciences Faculty, University of Campinas (UNICAMP), Tessália Vieira de Camargo, Campinas, Brazil; 4 MD, PhD. Department of Pathology, Medical Sciences Faculty, University of Campinas (UNICAMP), Tessália Vieira de Camargo, Campinas, Brazil

## Abstract

The synchronous occurrence of two different neoplasias is an uncommon event, which may arise between tumors originating in the same organ or in cancer-to-cancer metastasis. We report a rare case of chronic lymphocytic leukaemia / small lymphocytic lymphoma associated with a cutaneous metastatic squamous cell carcinoma in a cervical lymph node. In the affected lymph node, it was observed an effacement of the normal architecture by neoplastic lymphocytes and it was noted the presence of neoplastic invasive epithelial islands. Immunohistochemical analysis demonstrated that lymphocytic proliferation was positive for CD20, CD5, CD23 and Kappa, and negative for CD3, CD10, Cyclin D1 and Lambda. The morphological and immunohistochemical profile lead to a phenotype of B-cell chronic lymphocytic leukaemia / small lymphocytic lymphoma. The epithelial cells were positive for CK5, thus rendering the diagnosis of synchronous metastatic cutaneous squamous cell carcinoma and chronic lymphocytic leukaemia/small lymphocytic lymphoma. Literature supports the poor prognosis in cases that present coexistence of squamous cell carcinoma and chronic lymphocytic leukaemia / small lymphocytic lymphoma. Thus, it is necessary to be aware about this unusual finding in order to provide specific treatment.

** Key words:**Chronic lymphocytic leukaemia, small lymphocytic lymphoma, squamous cell carcinoma, metastasis.

## Introduction

The coexistence of two different neoplasias is a rare event, which may arise between tumors originating in the same organ or in cancer-to-cancer metastasis (1,2). In regard to cancer-to-cancer metastasis, lymphoma seems to be strongly related to metastasis by a secondary malignancy (3). In the head and neck region, lymphoma and squamous cell carcinoma (SCC) are common neoplasias, so simultaneous occurrence may be an eventual finding (4). In relation to chronic lymphocytic leukaemia (CLL) / small lymphocytic lymphoma (SLL), patients have susceptibility to develop second malignancies in 25% of the cases. To the best of our knowledge, only 12 cases of concomitant squamous cell carcinoma and CLL/SLL have been reported in the English-literature so far. These cases in general are more susceptible to recurrence, metastasis and death (5).

Herein, we report a rare case of CLL/SLL associated with an aggressive metastatic SCC. We also compared the characteristics of our case with previous reports of the literature.

## Case Report

**Figure 1:**
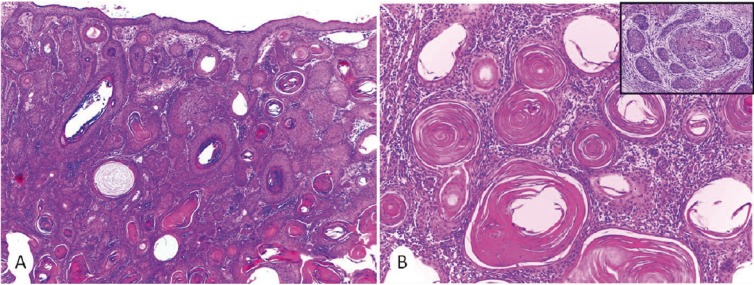
Histopathological features of primary cutaneous squamous cell carcinoma. A,B) Well differentiated squamous cell carcinoma with significant number of keratin pearls. Perineural invasion was found [inset] (H&E, A x40, B x100, inset x40).

A 71 year-old male presented with a papular, invasive, hyperkeratotic, desquamative and erythematous lesion measuring 1 cm in greater diameter in the left malar region of the skin. Concerning his medical history, he was diagnosed with chronic lymphocytic leukaemia / small lymphocytic lymphoma (CLL/SLL) three years ago, and was submitted to a chemotherapy regimen with fludarabine and cyclophosphamide. The cutaneous lesions was biopsied and microscopic features were characterized by an invasive growth downward the dermis. Cytologically, it was observed epithelial neoplastic cells with glassy eosinophilic cytoplasm, intercellular bridges, sometimes arranged in a concentric fashion with keratin pearls. The stroma was desmoplastic with chronic inflammatory infiltrate, and perineural invasion was identified (Fig. 1). These features lead to the diagnosis of cutaneous squamous cell carcinoma (SCC). After the diagnosis, he had the rest of the cutaneous lesion removed.

**Figure 2:**
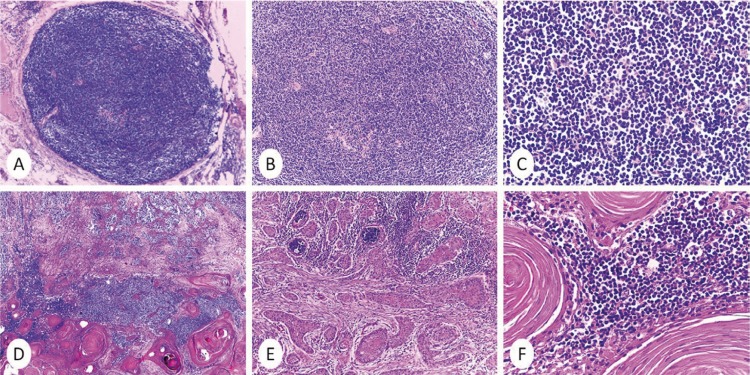
Histopathological features of synchronous metastatic cutaneous squamous cell carcinoma and chronic lymphocytic leukaemia/small lymphocytic lymphoma in lymph nodes. A) Diffuse effacement of lymph node by a proliferation of small lymphocytes. B,C) Monotonous population of small lymphocytes with round nuclei. D-F) Coexistence of well differentiated squamous cell carcinoma and chronic lymphocytic leukaemia/small lymphocytic lymphoma. (H&E, A-B x40, C x400, D-F x 40, F- x400).

Eight months later, the patient returned and it was observed a clinical involvement of the cervical lymph nodes, which had a hard consistency, adherence to deep tissues and measured up to 2.5 cm. A neck dissection was performed. Eight out of the 79 examined lymph nodes presented an effacement of the normal architecture by mature, small and uniform lymphocytes with a narrow border of cytoplasm and a densely-stained nucleus. Additionally, it was identified the presence of neoplastic invasive epithelial islands, with significant keratin pearls production. Besides, the epithelial clusters were infiltrating the connective tissue (Fig. 2).

**Figure 3:**
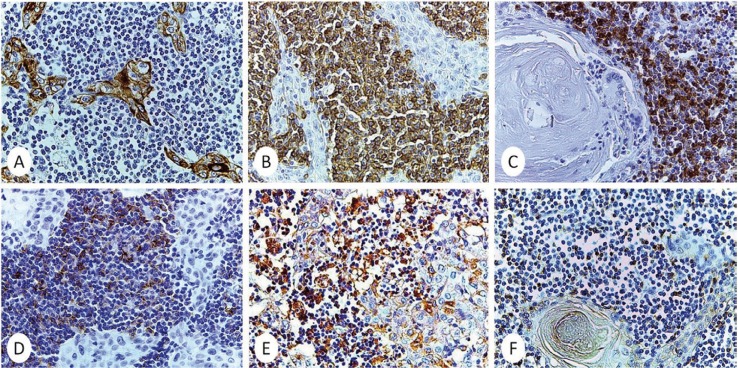
Immunohistochemical features of synchronous metastatic cutaneous squamous cell carcinoma and chronic lymphocytic leukaemia/small lymphocytic lymphoma in lymph nodes. A) Epithelial islands of SCC express CK5. B-E) The neoplastic lymphocytes express CD20 B), CD5 C), CD23 D) and Kappa E). Only scattered T lymphocytes are positive for CD3 F). (Immunoperoxidase, A-F x400).

An immunohistochemical assessment was performed with the antibodies listed in  Table 1. The epithelial cells were positive for CK5. In relation to neoplastic lymphoid cells it was observed positivity for CD20, CD5, CD23 and Kappa. CD3, CD10, Cyclin D1 and Lambda were negative. CD3 was only identified in reactive T lymphocytes (Fig. 3). The proliferative index with Ki-67 was 10%. The morphological and immunohistochemical profile were sufficient to render a diagnosis of synchronous metastatic cutaneous squamous cell carcinoma and chronic lymphocytic leukaemia/small lymphocytic lymphoma in a cervical lymph node.

**Table 1:**
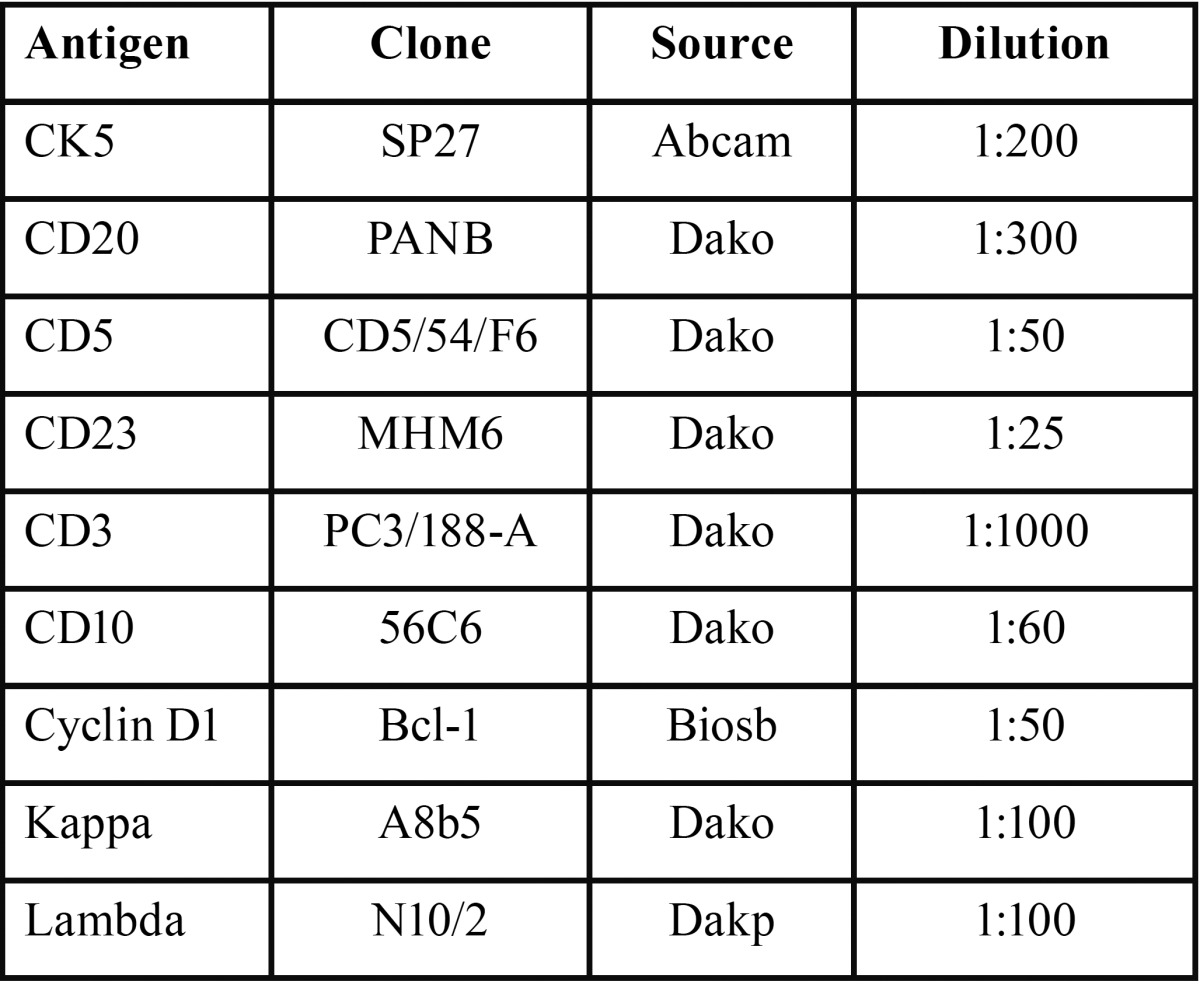
Antibodies used for immunohistochemistry in the case of synchronous metastatic cutaneous squamous cell carcinoma and chronic lymphocytic leukaemia/small lymphocytic lymphoma in lymph nodes.

The patient was submitted to radiotherapy and chemotherapy. In the last evaluation it was noticed absence of skin lesion as well as negative palpable lymph nodes. The patient is still under follow-up.

## Discussion

We described a case of a patient previously diagnosed with CLL/SLL who developed cutaneous SCC, which metastasized to lymph nodes affected by CLL/SLL. In Western countries, CLL/SLL is the most common adult leukemia and affects mainly males (6,7). Although its etiology remains unclear, genetic factors have been proposed as carcinogenic agents (6,8). CLL/SLL is featured by a clonal proliferation of mature and frequently CD5+ B- cells within bone marrow, blood, spleen and lymph nodes (6). Microscopically, the cells express CD5, CD19, CD23, and CD20 (9). The differential diagnosis included mantle cell lymphoma, follicular lymphoma and marginal-zone lymphoma (10). The immunohistochemical profile of the lymphoproliferative disorder in the present case was in accordance with the diagnosis of CLL/SLL.

The pathogenesis of secondary tumors associated with CLL/SLL patients is unknown, but some theories have been proposed such as immune dysfunction related to CLL/SLL (11), genetic susceptibility, age, exposure to common carcinogenic agents (12), mutagenic effects of chemotherapy and radiotherapy (13). Although the exactly mechanism underlying lymphoma-associated tumor is still unclear, we believe that our patient developed a metastatic cutaneous SCC as a secondary tumor related to the previously diagnosed lymphoproliferative disorder. The development of SCC in our patient is in accordance with a study that documented a spectrum of cutaneous conditions in patients with CLL/SLL, which included SCC as one of the most common lesions (14). In addition, the coexistence of SCC with CLL/SLL in the same lymph node is a rare event based on studies which concluded that 5% of patients with head and neck cancer presented unexpected findings in neck dissection, such as malignant tumors (15).

 Table 2 presents the clinical characteristic of previously reported cases of synchronous metastatic cutaneous squamous cell carcinoma and chronic lymphocytic leukaemia/small lymphocytic lymphoma in lymph nodes. In regard to age, the range was 44 to 82 years (mean, 72 years). All patients were male. Most of the sites affected by SCC were the skin of the head. From the available data, the interval between the diagnosis of CLL/SLL and first SCC was 6 years. According to the diagnosis of the simultaneous occurrence of SCC and CLL/SLL, it was mainly based on neck dissection (3,5,12,16-20). Hence, the clinical characteristics of our case corresponded to previously reported features in the literature.

**Table 2:**
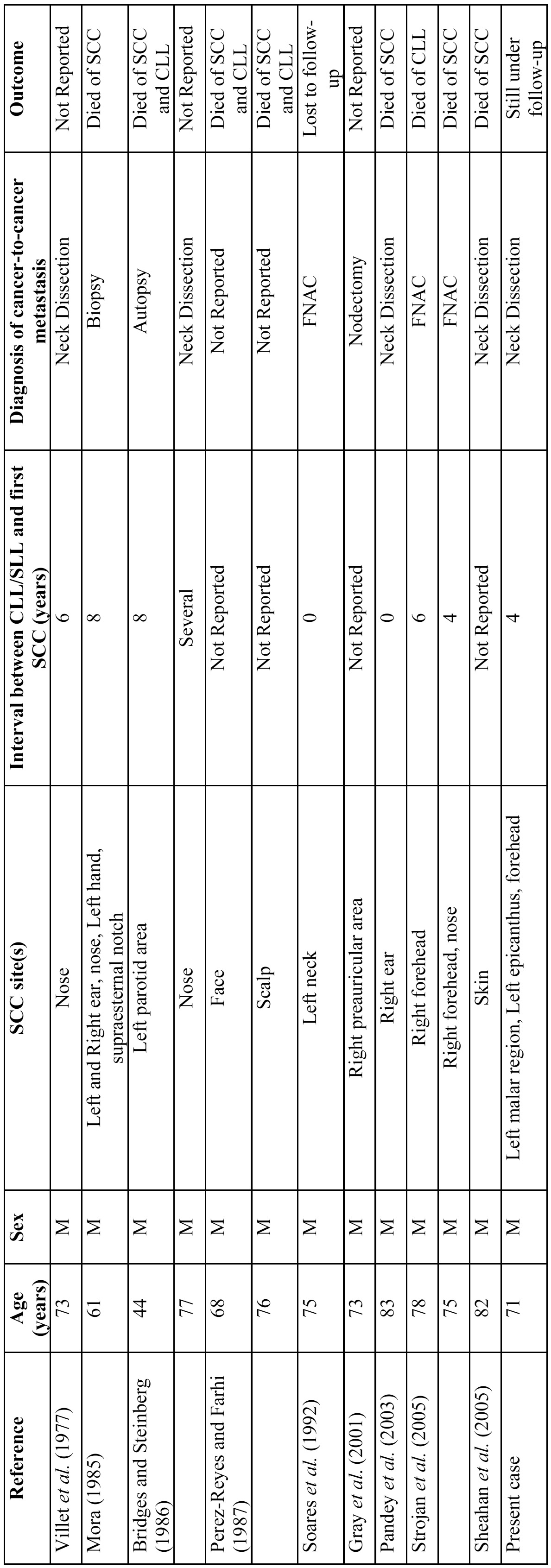
Clinical characteristic of previously reported cases of synchronous metastatic cutaneous squamous cell carcinoma and chronic lymphocytic leukaemia/small lymphocytic lymphoma in lymph nodes.

According to the literature, patients with CLL/SLL are prone to develop SCC with an aggressive biological behavior, featured by recurrence and extensive metastasis (18,21). The clinical behavior of SCC in our patient corresponds to those observations reported in the literature. In addition, the information about the outcome of  Table 2, showed that most of the patients died of SCC and/or CLL (3,5,12,16-20). Nevertheless, our patient stills alive without evidence of recurrence or metastasis.

In conclusion, the occurrence of CLL/SLL may be related to second malignancies, such as cutaneous SCC. The coexistence of metastatic cutaneous SCC and CLL/SLL in a lymph node is a rare event, which generally results in a poor prognosis. Therefore, it is necessary to be aware about this unusual finding as SCC and CLL/SLL require specific treatment.

## References

[1] Murthaiah P, Truskinovsky AM, Shah S, Dudek AZ (2009). Collision tumor versus multiphenotypic differentiation: a case of carcinoma with features of colonic and lung primary tumors. Anticancer Res.

[2] Binello E, Bederson JB, Kleinman GM (2010). Hemangiopericytoma: collision with meningioma and recurrence. Neurol Sci.

[3] Flezar MS, Prevodnik VK, Kirbis IS, Strojan P (2006). Cutaneous squamous cell carcinoma metastatic to chronic lymphocytic leukaemia: Diagnostic potential of fine needle aspiration cytology. Cytopathology.

[4] Kakarala K, Sadow PM, Emerick KS (2010). Cervical lymph node collision tumor consisting of metastatic squamous cell carcinoma and B-cell lymphoma. Laryngoscope.

[5] Perez-Reyes N, Farhi DC (1987). Squamous cell carcinoma of head and neck in patients with well-differentiated lymphocytic lymphoma. Cancer.

[6] Rozman C, Montserrat E (1995). Chronic lymphocytic leukemia. N Engl J Med.

[7] Chiorazzi N, Rai KR, Ferrarini M (2005). Chronic lymphocytic leukemia. N Engl J Med.

[8] Catovsky D (1997). The search for genetic clues in chronic lymphocytic leukemia. Hematol Cell Ther.

[9] Matutes E, Owusu-Ankomah K, Morilla R, Garcia Marco J, Houlihan A, Que TH (1994). The immunological profile of B-cell disorders and proposal of a scoring system for the diagnosis of CLL. Leukemia.

[10] Chang CC, Rowe JJ, Hawkins P, Sadeghi EM (2003). Mantle cell lymphoma of the hard palate: a case report and review of the differential diagnosis based on the histomorphology and immunophenotyping pattern. Oral Surg Oral Med Oral Pathol Oral Radiol Endod.

[11] Buell JF, Hanaway MJ, Thomas M, Alloway RR, Woodle ES (2005). Skin cancer following transplantation: the Israel Penn International Transplant Tumor Registry experience. Transplant Proc.

[12] Pandey U, Naraynan M, Karnik U, Sinha B (2003). Carcinoma metastasis to unexpected synchronous lymphoproliferative disorder: report of three cases and review of literature. J Clin Pathol.

[13] Rashid K, Ng R, Mastan A, Sager D, Hirschman R (2005). Accelerated growth of skin carcinoma following fludarabine therapy for chronic lymphocytic leukemia. Leuk Lymphoma.

[14] Agnew KL, Ruchlemer R, Catovsky D, Matutes E, Bunker CB (2004). Cutaneous findings in chronic lymphocytic leukaemia. Br J Dermatol.

[15] Sheahan P, Hafidh M, Toner M, Timon C (2005). Unexpected findings in neck dissection for squamous cell carcinoma: incidence and implications. Head Neck.

[16] Villet WT, Staples WG, Ge’taz EP (1977). Squamous cell carcinoma metastasis to lymph nodes in chronic lymphatic leukemia. S Afr Med J.

[17] Mora RG (1985). Metastatic squamous cell carcinoma of the skin occurring in a lymphomatous lymph node. J Am Acad Dermatol.

[18] Bridges N, Steinberg JJ (1986). Aggressive squamous cell carcinoma of the skin after chronic lymphocytic leukemia. J Surg Oncol.

[19] Soares FA, Potenciano O, Saldanha JC, Laemmel A (1992). Fine needle aspiration of squamous cell carcinoma of the skin metastatic to the site of leukemic lymphadenopathy. Acta Cytol.

[20] Gray Y, Robidoux HJ, Farrell DS, Robinson-Bostom L (2001). Squamous cell carcinoma detected by high-molecularweight cytokeratin immunostaining mimicking atypical fibroxanthoma. Arch Pathol Lab Med.

[21] Hartley BE, Searle AE, Breach NM, Rhys-Evans PH, Henk JM (1996). Aggressive cutaneous squamous cell carcinoma of the head and neck in patients with chronic lymphocytic leukemia. J Laryngol Otol.

